# Supplementary material for: Do family and maternal background matter? A multilevel approach to modelling mental health status of Australian youth using longitudinal data
Source: PLoS One. 2022 Apr 26;17(4):e0267191. doi: 10.1371/journal.pone.0267191 (PMC9041811; doi:10.1371/journal.pone.0267191)
Supplement: S1 Appendix — (DOCX) [file pone.0267191.s001.docx]

**S1 Appendix**

Variable description in HILDA for financial shock and life event shock

| Variables used to construct financial shock | | |
| --- | --- | --- |
| Sl | Variable name | Variable description |
| 1. | _fiprbeg | Could not pay electricity, gas or telephone bills on time |
| 2. | _fiprbmr | Could not pay the mortgage or rent on time |
| 3. | _fiprbps | Pawned or sold something |
| 4. | _fiprbwm | Went without meals |
| 5. | _fiprbuh | Was unable to heat home |
| 6. | _fiprbfh | Asked for financial help from friends or family |
| 7. | _fiprbwo | Asked for help from welfare/community organisations |
| Variables used to construct life event shock | | |
| 1. | _leins | Serious personal injury/illness |
| 2. | _leinf | Serious injury/illness to family member |
| 3. | _ledsc | Death of spouse or child |
| 4. | _ledrl | Death of close relative/family member |
| 5. | _ledfr | Death of a close friend |
| 6. | _levio | Victim of physical violence |
| 7. | _lepcm | Victim of a property crime |
| 8. | _lejls | Detained in jail |
| 9. | _lejlf | Close family member detained in jail |
| 10. | _lefrd | Fired or made redundant |
| 11. | _ledhm | A weather-related disaster (flood, bushfire, cyclone) damaged or destroyed your home |
