# Supplementary material for: Do family and maternal background matter? A multilevel approach to modelling mental health status of Australian youth using longitudinal data
Source: PLoS One. 2022 Apr 26;17(4):e0267191. doi: 10.1371/journal.pone.0267191 (PMC9041811; doi:10.1371/journal.pone.0267191)
Supplement: Attachment — Submitted filename: PONE-D-21-14569_reviewer-RAH.pdf [file pone.0267191.s002.pdf]

PLOS ONE

Does maternal background matter? A multilevel approach to modelling mental health status of Australian youth using longitudinal data

--Manuscript Draft--

**Manuscript Number:**	PONE-D-21-14569
**Article Type:**	Research Article
**Full Title:**	Does maternal background matter? A multilevel approach to modelling mental health status of Australian youth using longitudinal data
**Short Title:**	maternal background and mental health
**Corresponding Author:**	Rubayyat Hashmi<br>AIUB: American International University Bangladesh<br>Dhaka, BANGLADESH
**Keywords:**	Equity, Parental Characteristics, Socioeconomic Status, Family Background, Mental Health
**Abstract:**	Purpose<br>Most previous research place great store on the influence of maternal background on child and adolescents mental health. However, age of onset studies indicates that the majority of the mental health disease prevalence occurs during the youth years. This study investigates the relationship of maternal background and individual circumstance on youth mental health status.<br>Method<br>Data from 975 participants and 4632 observations of aged cohort 15 to 19 years in the Household, Income and Labour Dynamics in Australia (HILDA) longitudinal study were followed for 10 years (2007-2017).<br>Results<br>The findings suggests that not all dimensions of maternal background (especially education) have impacts on youth mental health. We found household income (AOR: 1.572, 95% CI: 1.017-2.43) and living arrangement (AOR: 1.586, 95% CI: 1.097-2.294) significantly increases mental disorder odds whereas maternal education or occupation fixed effects were not significant. Individual level circumstances have much stronger impact on youth mental health. We found financial shock (AOR: 1.412, 95% CI: 1.277-1.561), life event shock (AOR: 1.157, 95% CI: 1.01-1.326), long term health conditions (AOR: 2.855, 95% CI: 2.042-3.99), smoking (AOR: 1.676, 95% CI: 1.162-2.416), drinking (AOR: 1.649, 95% CI: 1.286-2.114) and being female (AOR: 2.021, 95% CI: 1.431-2.851) have significant deteriorating effects on youth mental health.<br>Conclusions<br>Our finding is in contrast to the majority of studies in the literature which give a preeminent role to maternal characteristics in child and youth mental health status. Mental health interventions should consider heterogeneity of adverse youth circumstances and health-related behaviours.
**Order of Authors:**	Rubayyat Hashmi
	Khorshed Alam
	Jeff Gow
	Sonja March
**Additional Information:**	
**Question**	**Response**
**Financial Disclosure**<br><br>Enter a financial disclosure statement that describes the sources of funding for the work included in this submission. Review the submission guidelines for detailed	NO - The funders had no role in study design, data collection and analysis, decision to publish, or preparation of the manuscript.

Powered by Editorial Manager® and ProduXion Manager® from Aries Systems Corporation

requirements. View published research articles from *PLOS ONE* for specific examples.<br><br>This statement is required for submission and **will appear in the published article** if the submission is accepted. Please make sure it is accurate.<br><br>**Unfunded studies**<br>Enter: *The author(s) received no specific funding for this work.*<br><br>**Funded studies**<br>Enter a statement with the following details:<br>• Initials of the authors who received each award<br>• Grant numbers awarded to each author<br>• The full name of each funder<br>• URL of each funder website<br>• Did the sponsors or funders play any role in the study design, data collection and analysis, decision to publish, or preparation of the manuscript?<br>• **NO** - Include this sentence at the end of your statement: *The funders had no role in study design, data collection and analysis, decision to publish, or preparation of the manuscript.*<br>• **YES** - Specify the role(s) played.<br><br>* typeset	
**Competing Interests**<br><br>Use the instructions below to enter a competing interest statement for this submission. On behalf of all authors, disclose any competing interests that could be perceived to bias this work—acknowledging all financial support and any other relevant financial or non-financial competing interests.<br><br>This statement is required for submission and **will appear in the published article** if the submission is accepted. Please make sure it is accurate and that any funding sources listed in your Funding Information later in the submission form are also declared in your Financial Disclosure	The authors have declared that no competing interests exist.

statement.<br><br>View published research articles from *PLOS ONE* for specific examples.<br><br>**NO authors have competing interests**<br><br>Enter: *The authors have declared that no competing interests exist.*<br><br>**Authors with competing interests**<br><br>Enter competing interest details beginning with this statement:<br><br>*I have read the journal's policy and the authors of this manuscript have the following competing interests: [insert competing interests here]*<br><br>* typeset	
**Ethics Statement**<br><br>Enter an ethics statement for this submission. This statement is required if the study involved:<br><br>• Human participants<br>• Human specimens or tissue<br>• Vertebrate animals or cephalopods<br>• Vertebrate embryos or tissues<br>• Field research<br><br>Write "N/A" if the submission does not require an ethics statement.<br><br>General guidance is provided below. Consult the submission guidelines for detailed instructions. **Make sure that all information entered here is included in the Methods section of the manuscript.**	This study did not require any ethics approval since it draws upon a secondary dataset. In addition, the Australian Government Department of Social Services (DSS) and Melbourne Institute of Applied Economic and Social Research at the University of Melbourne disseminate information in full compliance with the regulation pertaining to the privacy of the participants.

Powered by Editorial Manager® and ProduXion Manager® from Aries Systems Corporation

**Format for specific study types**<br><br>**Human Subject Research (involving human participants and/or tissue)**<br>• Give the name of the institutional review board or ethics committee that approved the study<br>• Include the approval number and/or a statement indicating approval of this research<br>• Indicate the form of consent obtained (written/oral) or the reason that consent was not obtained (e.g. the data were analyzed anonymously)<br><br>**Animal Research (involving vertebrate animals, embryos or tissues)**<br>• Provide the name of the Institutional Animal Care and Use Committee (IACUC) or other relevant ethics board that reviewed the study protocol, and indicate whether they approved this research or granted a formal waiver of ethical approval<br>• Include an approval number if one was obtained<br>• If the study involved *non-human primates*, add *additional details* about animal welfare and steps taken to ameliorate suffering<br>• If anesthesia, euthanasia, or any kind of animal sacrifice is part of the study, include briefly which substances and/or methods were applied<br><br>**Field Research**<br><br>Include the following details if this study involves the collection of plant, animal, or other materials from a natural setting:<br>• Field permit number<br>• Name of the institution or relevant body that granted permission	
**Data Availability**<br><br>Authors are required to make all data underlying the findings described fully available, without restriction, and from the time of publication. PLOS allows rare exceptions to address legal and ethical concerns. See the PLOS Data Policy and FAQ for detailed information.	No - some restrictions will apply

Powered by Editorial Manager® and ProduXion Manager® from Aries Systems Corporation

A Data Availability Statement describing where the data can be found is required at submission. Your answers to this question constitute the Data Availability Statement and **will be published in the article**, if accepted.<br><br>**Important:** Stating 'data available on request from the author' is not sufficient. If your data are only available upon request, select 'No' for the first question and explain your exceptional situation in the text box.<br><br>Do the authors confirm that all data underlying the findings described in their manuscript are fully available without restriction?	
**Describe where the data may be found in full sentences. If you are copying our sample text, replace any instances of XXX with the appropriate details.**<br><br>• If the data are **held or will be held in a public repository**, include URLs, accession numbers or DOIs. If this information will only be available after acceptance, indicate this by ticking the box below. For example: *All XXX files are available from the XXX database (accession number(s) XXX, XXX.*).<br>• If the data are all contained **within the manuscript and/or Supporting Information files**, enter the following: *All relevant data are within the manuscript and its Supporting Information files.*<br>• If neither of these applies but you are able to provide **details of access elsewhere**, with or without limitations, please do so. For example:<br><br>*Data cannot be shared publicly because of [XXX]. Data are available from the XXX Institutional Data Access / Ethics Committee (contact via XXX) for researchers who meet the criteria for access to confidential data.*<br><br>*The data underlying the results presented in the study are available from (include the name of the third party*	The data is available from the National Centre for Longitudinal Data (NCLD) of DSS for researchers of approved organisations who meet the criteria for access to confidential data.

and contact information or URL).<br>• This text is appropriate if the data are owned by a third party and authors do not have permission to share the data.<br><br>* typeset	
Additional data availability information:	

Manuscript
Click here to access/download;Manuscript;Manuscript_plos1_210503.docx

Does maternal background matter? A multilevel approach to modelling mental health status of Australian youth using longitudinal data

Rubayyat Hashmi[1]
Assistant Professor
Dept. of Economics, American International University-Bangladesh (AIUB)
408/1, Kuratoli, Khilkhet, Dhaka 1229, Bangladesh
Email: rubayyat@gmail.com ; and
School of Business, Faculty of Business, Education, Law & Arts
University of Southern Queensland, Toowoomba, Queensland 4350 Australia
http://orcid.org/0000-0002-5953-0650

Khorshed Alam, PhD
Professor (Economics)
Centre for Health Research
and
School of Commerce, Faculty of Business, Education, Law & Arts
University of Southern Queensland, Toowoomba, Queensland 4350 Australia
Ph: (+617) 4631 1291, Fax: (+617) 4631 5594 Email: Khorshed.Alam@usq.edu.au
http://orcid.org/0000-0003-2232-0745

Jeff Gow, PhD
Professor (Economics)
Centre for Health Research
and
School of Commerce, Faculty of Business, Education, Law & Arts
University of Southern Queensland, Toowoomba, Queensland 4350 Australia
Ph: (+617) 4631 2617, Fax: (+617) 4631 5594 Email: Jeffrey.Gow@usq.edu.au
and
School of Accounting, Economics and Finance, University of KwaZulu-Natal, Durban 4000, South Africa
http://orcid.org/0000-0002-5726-298X

Sonja March, PhD
Professor (Psychology)
Centre for Health Research
and
School of Psychology and Counselling, Faculty of Health, Engineering and Sciences
University of Southern Queensland, Springfield, Queensland 4300 Australia
Ph: (+617) 3470 4434, Email: Sonja.March@usq.edu.au
https://orcid.org/0000-0001-8425-7126

[1] Corresponding author

Abstract

Purpose

Most previous research place great store on the influence of maternal background on child and adolescents mental health. However, age of onset studies indicates that the majority of the mental health disease prevalence occurs during the youth years. This study investigates the relationship of maternal background and individual circumstance on youth mental health status.

Method

Data from 975 participants and 4632 observations of aged cohort 15 to 19 years in the Household, Income and Labour Dynamics in Australia (HILDA) longitudinal study were followed for 10 years (2007-2017).

Results

The findings suggests that not all dimensions of maternal background (especially education) have impacts on youth mental health. We found household income (AOR: 1.572, 95% CI: 1.017-2.43) and living arrangement (AOR: 1.586, 95% CI: 1.097-2.294) significantly increases mental disorder odds whereas maternal education or occupation fixed effects were not significant. Individual level circumstances have much stronger impact on youth mental health. We found financial shock (AOR: 1.412, 95% CI: 1.277-1.561), life event shock (AOR: 1.157, 95% CI: 1.01-1.326), long term health conditions (AOR: 2.855, 95% CI: 2.042-3.99), smoking (AOR: 1.676, 95% CI: 1.162-2.416), drinking (AOR: 1.649, 95% CI: 1.286-2.114) and being female (AOR: 2.021, 95% CI: 1.431-2.851) have significant deteriorating effects on youth mental health.

Conclusions

Our finding is in contrast to the majority of studies in the literature which give a preeminent role to maternal characteristics in child and youth mental health status. Mental health interventions should consider heterogeneity of adverse youth circumstances and health-related behaviours.

Keywords: Equity, Parental Characteristics, Socioeconomic Status, Family Background, Mental Health

1. Introduction

Social gradients in physical and mental health status exist and good mental health is not equally distributed [1-3]. Understanding the determinants of socioeconomic inequality is important for policy makers and researchers alike. While socioeconomic inequalities in adult mental health dominates current research, a growing body of literature currently points to the importance of maternal background on children and adolescents’ mental health [4-6]. Accumulating evidence also suggests that lower socioeconomic status (SES) is an important marker of mental health problems in children and adolescents [7-9].

Although child and adolescent periods appear to be emerging points for mental disorders, age of onset (AOO) studies have identified that the majority of mental disorder incidence occurs at the early stages of youth, particularly when young people transition to adulthood [10, 11]. The problem in the literature relating to the influences of maternal background and childhood circumstances on mental health status is that the age bands in these studies are broad, obscuring the stages of youth by either younger youths being included with ‘children and adolescents’ (e.g. age 1-18 years) or older youths being included with ‘adults’ (e.g. 15-64 years) [8, 12, 13]. The circumstances experienced by individuals in their childhood and adolescent period are certainly much different than the period when they are transitioning to youth and adulthood. Thus, the impact of maternal background on this transitioning phase on an individual’s mental health outcome is not clear and may very well be different.

In this paper, we tried to address this issue by selecting a 15-19 years age cohort and following the cohort for ten years (up to six measurement points) to investigate the impact of youth circumstances on mental health outcomes. Although significant advances have been made in our understanding of the impact of maternal background on childhood mental health status, considerable knowledge gaps still exist. For instance, we do not understand how different attributes that constitutes maternal social class variations (such as mothers education, income or occupational status) contributes to the variation in youth mental status or how such inequalities evolve over time. Little is known about the variability of individual level and social class level characteristics on mental health outcome inequalities for youth and young people.

Thus, the primary goal of this paper is to fill this knowledge gap and attempt to provide a link between prior studies on childhood and adult mental health inequalities. In addition, the focus on Australian youth complements existing US, UK or European studies on youth mental health inequalities. Our study extends the literature to another developed country with different social welfare system and norms that provide different perspectives on mental health equity issues. We also extend the scope of our research by using improved modelling techniques, for example, utilising multi-level modelling to assess mental health outcomes, which is another major contribution of this study.

2. Methods

2.1 Data source

All our analyses are based on sample data from the Household, Income and Labour Dynamics in Australia (HILDA) panel survey[14]. This nationally representative household survey has been carried out annually from 2001 through 2018 (waves 1-18). It interviews and subsequently reinterviews all members aged 15 years and over of the same selected household every year. More than 30,000 individuals (40,000+ enumerated) have participated in the survey over the years and on average 15,000 individuals have been interviewed every year. A 90% wave on wave response rates of HILDA survey are comparable with other large longitudinal surveys like the British Household Panel Study (BHPS) or Panel Study of Income Dynamics (PSID). Details of HILDA sample design, survey response rates and attrition rates can be found elsewhere [15].

<Insert Figure 1 here>

2.2 Inclusion criteria of the samples

For the purpose of this study, we limit the sample to young Australians aged 15-19 years (late adolescent period) at the baseline wave (wave 7) and then followed the participants for 10 years (up to six measurement points) which covers youth (20-24 years) and transition to adulthood phase (25-29 years) in the follow up. We chose to start from wave 7, because HILDA survey did not start to collect Kessler Psychological Distress Scale (K10) scores (our main outcome of interest) in earlier waves and it provides the score subsequently in every odd wave (every two years) thereafter. Thus we constructed

an unbalanced panel data using wave 7, 9, 11, 13, 15 and 17. To be included in the analyses, the participants had to be interviewed in the baseline wave 7 and has to appear in at least one of the follow-up waves. Our final sample contains 975 participants across the six waves with a total of 4,632 observations. The 15-19 age cohort was thus followed up to 25-29 years with an average of 5.18 observations per person. The participant flow into the sample is shown in Figure 1.

2.3 Outcome variable, exposure variables and other co-variates

This study uses the Kessler Psychological Distress Scale (K10) as the measure of mental health outcomes and is the main dependent variable for analyses[16]. In clinical practice, the scale is used to assess the likelihood of having a mental disorder; for example, a person with a score of 10-15 has a low risk of having a mental disorder whereas a person with a score of 20-24 is likely to have a mild mental disorder, a score of 25-30 would indicate a likely moderate mental disorder and a person with a score of 30-50 is likely to have a severe mental disorder[17]. In the analyses, we use a dichotomous K10 variable (where a score of greater than 20 depict the likelihood of a mental disorder) as measures of our dependent variable for mental health performance [18].

Following Roemer's equality of opportunity theory [19, 20] we classify all our exposure variables into two types: i) circumstances category and ii) effort category. The theory of equality of opportunity revolves around the goal of compensating for 'negative' circumstances (such as parental background) on health outcomes while disregarding the health inequalities generated by effort category variables (such as lifestyle or health habits) that can be attributed to the behaviour of an individual. We use the biological mothers' education level and occupational status, household income and family living arrangements (whether the participant lived with both parents at the age of 14 years old) to determine the maternal background status as a group level characteristic of the circumstances category. We define maternal education level as low if the highest qualification level obtained by the mother is secondary level or lower. We use the Australian Socioeconomic Index 2006 (AUSEI06) occupational status scale as the measure of the occupational status of mother [21]. We assign occupational status as low if the value range falls in the lowest quintile. Similarly, we assign household income as low if the equivalised household income range falls in the lowest quintile. Using household income, family living

arrangement, maternal education and occupational status we have constructed 16 (2x2x2x2) different types of maternal background history groups for the multilevel analyses.

We use the number of financial shocks, number of life event shocks and long term health conditions in the individual level circumstances category[12]. The number of financial shock variable shows the number of adverse financial events the study participant has experienced (for example: went without meals or asked for financial help from friends or family). Similarly, life event shock variable shows the number of life events related to grief, loss or injury the study participant has suffered (for example: death of a family member or serious personal injury). The list of events that constitutes financial and life event shocks are given in the appendix. We use negative health habits such as being obese (as a proxy of unhealthy eating and lack of exercising), being a daily smoker and regular drinker (drinks more than four standard drink/day), and positive health habits such as being an active member of a sporting/hobby/community based club or association as an effort type of variables. This study also included gender and rural residency as demographic covariates in the analyses on the basis of past literature [22]. In addition, we construct our time variable by setting zero at the baseline wave 7 and subsequently adding two for each additional measurement point (since between wave time period is two years and there are up to six measurement points) to get a ten year follow-up at wave 17 (t= 0,2,4,6,8, and 10).

2.4 Statistical Analyses

The authors constructed an unbalanced longitudinal data set of the youth cohort by linking an individual's record who participated in the baseline (wave 7) at age 15-19 years and in one of the follow-up waves (9, 11, 13, 15 and 17). Descriptive statistics and mental health opportunity profile were summarized to understand the impact of maternal background characteristics on youth mental health. Visual trends of psychological distress scale were analysed for group level characteristics. Traditional single level regression analysis such as logistic regression model only assumes fixed-effect impacts of dependent variables and does not allow for random effects of intercepts and slopes for individual and group level characteristics. However, data structure can be nested or clustered by some observable characteristics that creates similarity between individuals and ignoring this phenomena can violate the

independence assumption of regression analysis. Multi-level models allow for a nested data structure and make it possible to study sources of variance at different levels of an outcome variable [23]. In our analyses, we used both single level logistic regression and multilevel logistic regression models. we have nested our data structure into three levels: i) time, ii) individual, and iii) maternal background history (a total of 16 different background history types; for example a background history type could be: household income: high, mothers education: low, mothers occupation: low and family living arrangement - whether not lived with both biological parents: yes.) We assigned unique id[illegible]iers for each group for the analysis. We control for individual fixed effects characteristics like circumstances and effort covariates in level 2 and group level fixed effects characteristics like various maternal background characteristics in level 3. All statistical analyses were conducted using Stata 15.

3. Results

3.1 Describing the sample

<Insert Table 1 here>

Table 1 displays the socio-demographic characteristics of the study population by mental health status. It can be seen that age groups do not vary significantly in mean K10 score both in the baseline wave and in all waves average. However, in our sample, males have lower average K10 score than females in both baseline wave and all waves average. Richer household income group has on average two-point lower K10 scores at baseline and approximately three points in all waves average. Those youth, who did not live with both biological parents at age 14, have two-point higher average K10 scores both in baseline and all waves average. Maternal education level does not indicate any significant difference in average scores between education groups. However, mothers with lower occupational status have approximately one point higher average scores. All groups have approximately six to seven points of standard deviation which indicates considerable variability at the individual level.

<Insert Table 2 here>

For a deeper understanding of maternal background groups, the mental health opportunity profile of the study participants are provided in Table 2. Depending upon household income, maternal education,

maternal occupation and living history arrangement of the participant, 16 types of background groups are identified. The groups are ranked in ascending order according to the average K10 score (lower score implies better mental health). Out of 16 groups, there are three groups with high risk level of developing a mental disorder. Three more groups also show a K10 average of more than 19 and sightly avoid entering into the high risk group. In addition, the high household income attribute has been clustered into lower rankings and vice versa. To further investigate, we plot the temporal evolution by the 16 maternal background types in Figure 2. The thick line (trend values varies between 15 and 25) shows that there also exist a lot of group level variability overtime in the average K10 scores. The trend analysis thus indicates both individual and group level variability and justifies analysing the data through a multi-level modelling approach.

<Insert Figure 2 here>

3.2 Regression analysis

The results of the regression models are in Table 3. Since, a single point change in the average K10 score might not mean anything unless it drives up into other risk categories Table 3 considers a dichotomous dependent variable (K10 ≥ 20 implies a higher risk of mental disorder) which measures risks through nonlinear estimation of odds ratios. The 'null' model results are shown in the first column. The 'null' model considers no explanatory variable and focuses just between and within individual variability. The random effect variances estimates for both maternal background level (level 3 σ^2_{v0} is 0.423 and SE is lower at 0.202) and individual level (level 2 σ^2_{u0} is 4.101 and SE is also much lower at 0.422) of the null model justifies the use of the multi-level approach. The second model in Table 3 shows the fixed effect logit estimates for comparison purpose. Unlike multilevel (ML) models, the logit does not have a random component and only shows fixed effects of the variables. To understand the maternal background variability, we do not consider the fixed effect of maternal background in the third model (Mixed 1 multilevel model). However, the final multilevel model (mixed 2) considers maternal background fixed effects. Individual fixed effects are considered in all models.

<Insert Table 3 here>

The individual level circumstances category variables are highly significant in all models. For example, exposure to an additional financial shock has a 1.4 times higher risk of having a mental illness than individuals who do not experience a shock (logit Adjusted Odds Ratio [AOR]: 1.321, 95% CI: 1.243-1.404; Mixed 1 AOR: 1.436, 95% CI: 1.298-1.589 and Mixed 2 AOR: 1.412, 95% CI: 1.277-1.561). Similarly, a single life event shock increases the risk of having mental disorder by 1.15 times higher (logit AOR: 1.156, 95% CI: 1.059-1.262; Mixed 1 AOR: 1.161, 95% CI: 1.013-1.331 and Mixed 2 AOR: 1.157, 95% CI: 1.01-1.326). This is considerable if you consider the possibility of experiencing multiple life events and financial shocks in a period. In addition, the study result also found that individuals who have long term health conditions are approximately 2.9 times highly likely to have a mental condition (logit AOR: 2.232, 95% CI: 1.853-2.688; Mixed 1 AOR: 2.934, 95% CI: 2.098-4.103 and Mixed 2 AOR: 2.855, 95% CI: 2.042-3.99).

The individual effort or lifestyle category variables such as ‘daily smoker’, ‘heavy drinker’ and ‘active membership of club or sporting activities’ are also significant in all models. Club activities have a positive effect on mental health (logit AOR: 0.651, 95% CI: 0.559-0.758; Mixed 1 AOR: 0.623, 95% CI: 0.487-0.797 and Mixed 2 AOR: 0.635, 95% CI: 0.496-0.812). On the contrary, negative habits such as smoking (logit AOR: 1.241, 95% CI: 1.018-1.512; Mixed 1 AOR: 1.801, 95% CI: 1.246-2.604 and Mixed 2 AOR: 1.676, 95% CI:1.162-2.416) and drinking (logit AOR: 1.344, 95% CI: 1.163-1.554; Mixed 1 AOR: 1.651, 95% CI: 1.288-2.117 and Mixed 2 AOR: 1.649, 95% CI: 1.286-2.114) have deteriorating effects on mental health. This study, however, did not find any significant association of being obese and mental health for the study cohort in all our models. In the case of demographic variables, the study found that women are twice as likely as men to have a mental disorder (logit AOR: 1.484, 95% CI: 1.286-1.712; Mixed 1 AOR: 2.063, 95% CI: 1.461-2.913 and Mixed 2 AOR: 2.021, 95% CI: 1.431-2.851). However, the ‘rural resident’ variable was found to be significant in only the logit estimate (AOR: 0.759, 95% CI: 0.593-0.97). In addition, the study found not significant association between the sample period (time variable) and mental disorder of the study cohort.

In our findings, individual-level fixed effects have much stronger impacts on mental health than maternal background characteristics. We found that only household income and parental living

arrangement (whether participants did not have the opportunity to live with both biological parents) were significant. Individuals who grew up in a poor household have approximately 1.6 times more likely to have mental disorder compared to youth who grew up in an affluent family (logit AOR: 1.258, 95% CI: 1.05-1.506; Mixed 2 AOR: 1.572, 95% CI: 1.017-2.43). Similarly, individuals who did not grow up with both biological parents in their childhood have approximately 1.6 times more likely to have mental disorder compared to the youths who grew up with both parents (logit AOR: 1.183, 95% CI: 1.017-1.376; Mixed 2 AOR: 1.586, 95% CI: 1.097-2.294). However, in our study, both mother's education and occupational status were not significant in any model. In addition, the random variances of maternal background in multilevel models were much lower compared to the null model (Null σ^2_{v0}: 0.423, 95% CI: 0.166-1.08 and Mixed 1 σ^2_{v0} : 0.078, 95% CI: 0.01-0.608). Indeed, the background variance disappears if fixed effect background characteristics are considered. Contrary to background random effects, individual level intercept variances are much larger (Null σ^2_{u0}: 4.101, 95% CI: 3.353-5.017, Mixed 1 σ^2_{u0} : 4.068 95% CI: 2.878-5.749 and Mixed 2 σ^2_{u0} : 4.116, 95% CI: 2.921-5.8). In summary, rather than the group level maternal backgrounds, the driving forces in mental health outcomes of the youths are the individual-level characteristics.

4. Discussion

The present study aimed to investigate the influence of group level maternal background characteristics and individual level circumstances-effort characteristics on the performance of youth mental health over time in Australia. For this purpose, the study sampled the 15-19 years cohort data from the long-running HILDA survey and followed 10 years for up to six measured points. Past research amassed substantial evidence in linking maternal education and occupation, with child's health outcomes [4, 6, 9]. However, contrary to this, we did not find any evidence linking youths' mental health with mother's education in any of our regression results. Perhaps, the thesis examined by Patrick W[illegible]n earlier research plays a role in this context. West argued that youth, in contrast to childhood, possess a process of equalisation which removes the influences of certain dimensions of family background differences (such as maternal education in our case) in youth mental health [24]. Few studies have explored this area, and further work is needed for the youth age groups. It is possible that as youth become more independent that the

influence of mothers' education becomes less important. We did, however, find significant impact of household income and family living arrangement on mental health performance of the youth. This impact is supported by other empirical literature [4, 6, 9, 25].

In order to investigate the underlying value judgement of individual effects, we followed equality of opportunity theory and categorised our variables into circumstances and effort groups [19, 20]. Our estimated results are consistent with the theory. We found that financial shocks, life event shocks and long term health conditions significantly deteriorate youth mental health condition. These findings are consistent with the adverse event literature [12, 26, 27]. In addition, we found that negative health habits such as smoking and drinking worsen mental health where as positive social habits such a club or sporting activities favours mental health, which is also in line with existing research [28]. Certainly, as youth become independent, the role of social relationships with those outside of families become particularly important in bolstering mental health.

One of the major contribution of this study is that we considered individual and group level variability through a multilevel modelling technique that other studies in the literature ignore. We found that there exists significant variability in individual level characteristics. In addition, individual level slope and intercepts also varied across time. However, compared with individual effects, the group level impact of maternal background characteristics did not vary. The implication of our finding is that, even though, some d[illegible]nsions of maternal background have significant influences, the impact of maternal background is much smaller than the individual effects such as financial and adverse life events, long-term health conditions, and health behaviour related activities (smoking and drinking habits).

Our results and findings have some interesting implications. Our findings stimulate discussion about the mechanism of maternal background linking the mental health childhood and adult cohorts. The findings suggest, more research is needed both in childhood and adult cohorts to further our understanding as to the impact of maternal background. Whilst maternal background may shape health in early childhood, its role in shaping youth health and mental health may not be so clear. On the other hand, there are number of factors that are clearly linked to youth mental health trajectories, including their physical health during ages 15-19. Policy makers might therefore be interested in implementing

health related behavioural interventions to promote both physical and mental health. Another observation of this study also suggests the importance of providing ongoing support to youth who have experienced financial and adverse life events in order to prevent long-term mental illness. This may include financial, care coordination and emotional support to manage the consequences of the adverse events in the short-term and trauma-informed psychological care in the long-term. Detailed research in the methodology and design of such interventions as well as estimation of the associated delivery costs of such program are needed.

Conclusions

In summary, our findings contribute to current knowledge by drawing attention to the lack of impact of maternal background on youth mental health. It is imperative that future research examines further the link of maternal background between younger and older age cohorts. The main strength of our study is the use of an equality of opportunity framework and multilevel modelling techniques to address critical questions on youth mental health in Australia. Policy-wise, mental health interventions should consider heterogeneity of adverse youth circumstances and health-related behaviours. This research will provide essential insights into how to improve such interventions.

Authors' contributions

RH conceptualised the study, conducted the data analysis and drafted the manuscript. KA, JG and SM offered advice, critical comments and edited the draft manuscript. All authors contributed to revisions of the manuscript and approved the final version of the manuscript prior to its submission.

Conflict of interest

The authors declare that they have no potential conflicts of interest with respect to the research, authorship, and/or publication of this article.

Ethics statement

This study did not require any ethics approval since it draws upon a secondary dataset. In addition, the Australian Government Department of Social Services (DSS) and Melbourne Institute of Applied

Economic and Social Research at the University of Melbourne disseminate information in full compliance with the regulation pertaining to the privacy of the participants.

Data availability

The data is available from the National Centre for Longitudinal Data (NCLD) of DSS for researchers of approved organisations who meet the criteria for access to confidential data.

Code availability

The programming code for analyses in the study can be made available upon request as determined by the authors.

References

1. Fryers T, Melzer D, Jenkins R. Social inequalities and the common mental disorders - A systematic review of the evidence. Social Psychiatry and Psychiatric Epidemiology. 2003;38(5):229-37. doi: 10.1007/s00127-003-0627-2.
2. Marmot M, Bell R. Fair society, healthy lives. Public Health. 2012;126:S4-S10. doi: https://doi.org/10.1016/j.puhe.2012.05.014.
3. Hashmi R, Alam K, Gow J, March S. Prevalence of Mental Disorders by Socioeconomic Status in Australia: A Cross-Sectional Epidemiological Study. American Journal of Health Promotion. 2020:0890117120968656. doi: 10.1177/0890117120968656.
4. Arroyo-Borrell E, Renart G, Saurina C, Saez M. Influence maternal background has on children's mental health. International Journal for Equity in Health. 2017;16(1):63. doi: 10.1186/s12939-017-0559-1.
5. Cui Y, Liu H, Zhao L. Mother's education and child development: Evidence from the compulsory school reform in China. Journal of Comparative Economics. 2019;47(3):669-92. doi: https://doi.org/10.1016/j.jce.2019.04.001.
6. Meyrose A-K, Klasen F, Otto C, Gniewosz G, Lampert T, Ravens-Sieberer U. Benefits of maternal education for mental health trajectories across childhood and adolescence. Social Science & Medicine. 2018;202:170-8. doi: https://doi.org/10.1016/j.socscimed.2018.02.026.
7. Koivusilta LK, Rimpelä AH, Kautiainen SM. Health inequality in adolescence. Does stratification occur by familial social background, family affluence, or personal social position? BMC Public Health. 2006;6:110-. doi: 10.1186/1471-2458-6-110. PubMed PMID: 16643660.
8. McLaughlin KA, Breslau J, Green JG, Lakoma MD, Sampson NA, Zaslavsky AM, et al. Childhood socio-economic status and the onset, persistence, and severity of DSM-IV mental disorders in a US national sample. Social Science & Medicine. 2011;73(7):1088-96. doi: https://doi.org/10.1016/j.socscimed.2011.06.011.
9. Reiss F. Socioeconomic inequalities and mental health problems in children and adolescents: A systematic review. Social Science & Medicine. 2013;90:24-31. doi: https://doi.org/10.1016/j.socscimed.2013.04.026.
10. Girolamo Gd, Dagani J, Purcell R, Cocchi A, McGorry PD. Age of onset of mental disorders and use of mental health services: needs, opportunities and obstacles. Epidemiology and Psychiatric Sciences. 2012;21(1):47-57. Epub 2011/12/13. doi: 10.1017/S2045796011000746.
11. Kessler RC, Amminger GP, Aguilar-Gaxiola S, Alonso J, Lee S, Üstün TB. Age of onset of mental disorders: a review of recent literature. Current Opinion in Psychiatry. 2007;20(4).
12. Hashmi R, Alam K, Gow J. Socioeconomic inequalities in mental health in Australia: Explaining life shock exposure. Health Policy. 2020;124(1):97-105. doi: https://doi.org/10.1016/j.healthpol.2019.10.011.
13. Reiss F, Meyrose A-K, Otto C, Lampert T, Klasen F, Ravens-Sieberer U. Socioeconomic status, stressful life situations and mental health problems in children and adolescents: Results of the German BELLA cohort-study. PLOS ONE. 2019;14(3):e0213700. doi: 10.1371/journal.pone.0213700.
14. The Household, Income and Labour Dynamics in Australia (HILDA) Survey, RESTRICTED RELEASE 18 (Waves 1-18) [Internet]. ADA Dataverse. 2020. Available from: http://dx.doi.org/10.26193/BBOTSM.
15. Summerfield M, Bright S, Hahn M, La N, Macalalad N, Watson N, et al. HILDA User Manual - Release 18. Australia: Melbourne Institute: Applied Economics and Social Research, University of Melbourne; 2019.
16. Kessler RC, Andrews G, Colpe LJ, Hiripi E, Mroczek DK, Normand SLT, et al. Short screening scales to monitor population prevalences and trends in non-specific psychological distress. Psychological Medicine. 2002;32(6):959-76. Epub 2002/09/26. doi: 10.1017/S0033291702006074.
17. Wooden M. Use of the Kessler Psychological Distress Scale in the HILDA Survey. Melbourne: Melbourne Institute of Applied Economic and Social Research, 2009.
18. Andrews G, Slade T. Interpreting scores on the Kessler Psychological Distress Scale (K10). Australian and New Zealand Journal of Public Health. 2001;25(6):494-7. doi: 10.1111/j.1467-842X.2001.tb00310.x.

19. Roemer JE. Equality of Opportunity. Cambridge, Mass.: Harvard University Press; 1998.
20. Roemer JE, Trannoy A. Equality of Opportunity: Theory and Measurement. Journal of Economic Literature. 2016;54(4):1288-332. doi: 10.1257/jel.20151206.
21. McMillan J, Beavis A, Jones FL. The AUSEI06: A new socioeconomic index for Australia. Journal of Sociology. 2009;45(2):123-49. doi: 10.1177/1440783309103342.
22. Silva M, Loureiro A, Cardoso G. Social determinants of mental health: A review of the evidence. Eur J Psychiatry. 2016;30(4):259-92.
23. Evans CR, Williams DR, Onnela J-P, Subramanian SV. A multilevel approach to modeling health inequalities at the intersection of multiple social identities. Social Science & Medicine. 2018;203:64-73. doi: https://doi.org/10.1016/j.socscimed.2017.11.011.
24. West P. Health inequalities in the early years: Is there equalisation in youth? Social Science & Medicine. 1997;44(6):833-58. doi: https://doi.org/10.1016/S0277-9536(96)00188-8.
25. Perales F, Johnson SE, Baxter J, Lawrence D, Zubrick SR. Family structure and childhood mental disorders: new findings from Australia. Social Psychiatry and Psychiatric Epidemiology. 2017;52(4):423-33. doi: 10.1007/s00127-016-1328-y.
26. Dalgard OS, Bjork S, Tambs K. Social support, negative life events and mental health. British Journal of Psychiatry. 1995;166(JAN.):29-34.
27. Schilling EA, Aseltine RH, Jr., Gore S. Adverse childhood experiences and mental health in young adults: a longitudinal survey. BMC Public Health. 2007;7:30-. doi: 10.1186/1471-2458-7-30. PubMed PMID: 17343754.
28. Buttery AK, Mensink GBM, Busch MA. Healthy behaviours and mental health: findings from the German Health Update (GEDA). Eur J Public Health. 2014;25(2):219-25. doi: 10.1093/eurpub/cku094.

Appendix A

Variable description in HILDA for financial shock and life event shock

Sl	Variable name	Variable description
		Variables used to construct financial shock
1.	_fiprbeg	Could not pay electricity, gas or telephone bills on time
2.	_fiprbmr	Could not pay the mortgage or rent on time
3.	_fiprbps	Pawned or sold something
4.	_fiprbwm	Went without meals
5.	_fiprbuh	Was unable to heat home
6.	_fiprbfh	Asked for financial help from friends or family
7.	_fiprbwo	Asked for help from welfare/community organisations
		Variables used to construct life event shock
1.	_leins	Serious personal injury/illness
2.	_leinf	Serious injury/illness to family member
3.	_ledsc	Death of spouse or child
4.	_ledrl	Death of close relative/family member
5.	_ledfr	Death of a close friend
6.	_levio	Victim of physical violence
7.	_lepcm	Victim of a property crime
8.	_lejls	Detained in jail
9.	_lejlf	Close family member detained in jail
10.	_lefrd	Fired or made redundant
11.	_ledhm	A weather related disaster (flood, bushfire, cyclone) damaged or destroyed your home

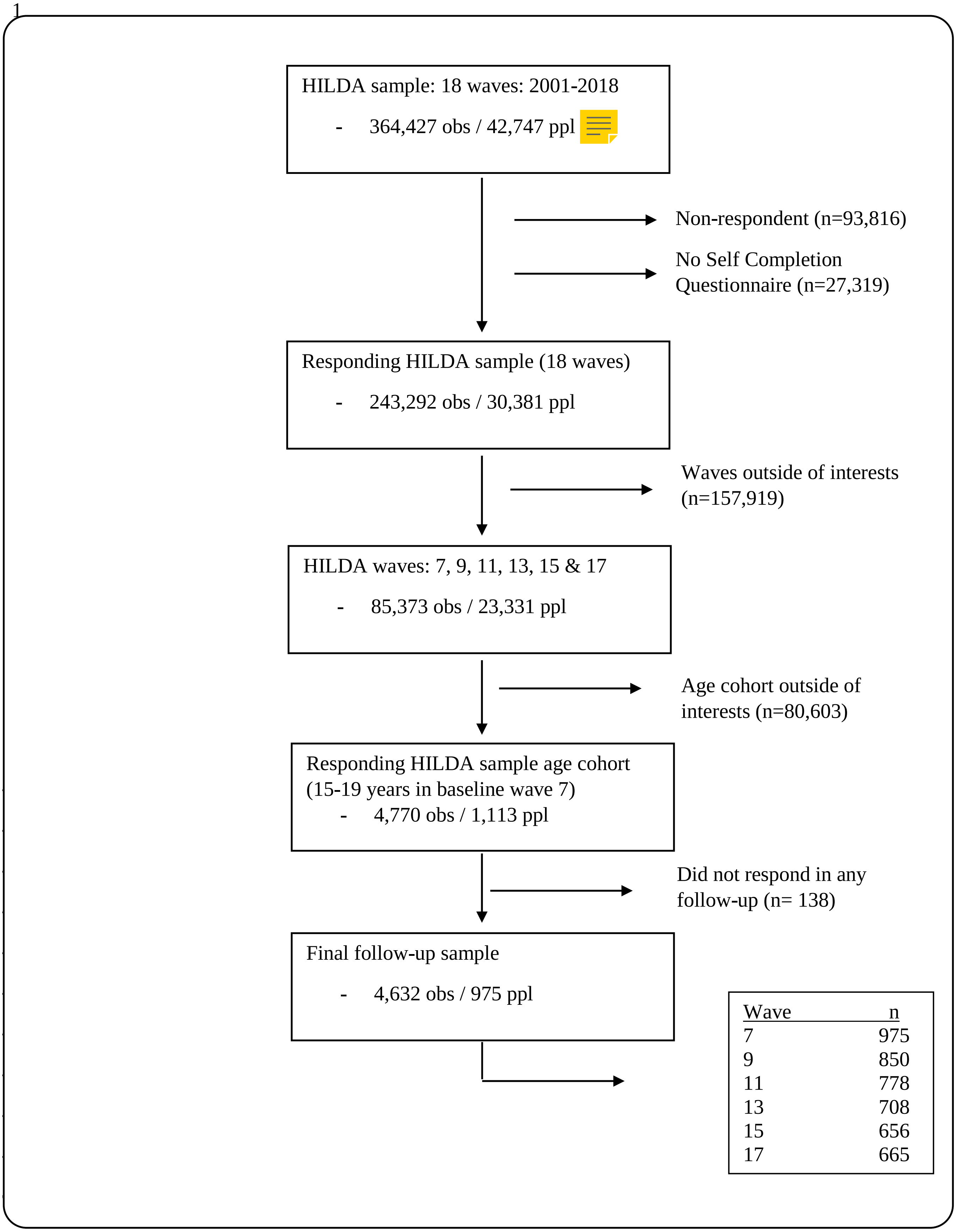

Figure 1: Participants flow into the sample

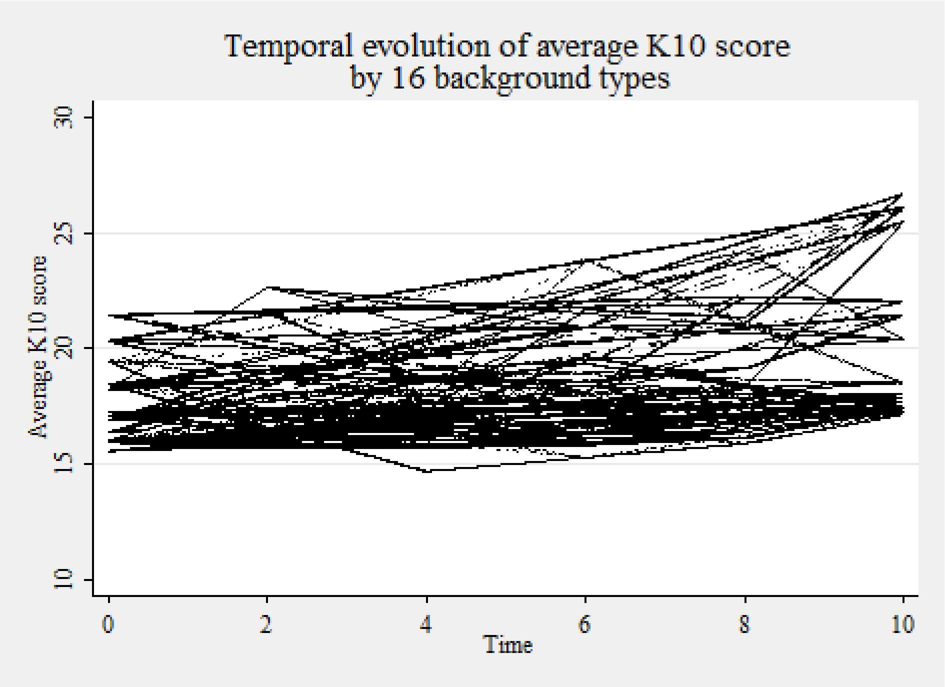

Figure 2: Temporal evolution of mental health Status (K10 score)

by background

Table 1 Socio-demographic characteristics of the study population by mental health status

	Baseline (wave 7)		All waves	
	**N (%)**	**K10 score Mean (std)**	**N (%)**	**K10 score Mean (std)**
**Gender**				
Male	465 (47.69)	15.76 (5.87)	2,109 (45.53)	16.39 (6.51)
Female	510 (52.31)	17.78 (6.92)	2,523 (54.47)	17.77 (7.25)
**Age**				
15 years	197 (20.21)	16.62 (6.84)	197 (4.25)	16.62 (6.84)
16 years	240 (24.62)	16.60 (6.29)	240 (5.18)	16.60 (6.29)
17 years	184 (18.87)	17.38 (6.43)	363 (7.84)	17.22 (6.74)
18 years	195 (20)	16.8 (6.26)	399 (8.61)	16.84 (6.43)
19 years	159 (16.31)	16.77 (6.89)	466 (10.06)	16.85 (6.79)
**HH Income group (Lowest quintile)**				
Low	222 (22.77)	18.37 (7.65)	931 (19.78)	19.34 (8.32)
High	753 (77.23)	16.36 (6.07)	3716 (80.22)	16.59 (6.46)
**Mother's Education (Low= secondary or lower)**				
Low	204 (20.92)	16.80 (6.67)	1759 (37.97)	17.71 (7.27)
High	771 (79.08)	16.80 (6.48)	2873 (62.03)	17.00 (6.87)
**Mother's occupational status (Lowest quintile)**				
Low	216 (22.15)	17.43 (7.12)	943 (20.36)	18.46 (7.88)
High	759 (77.85)	16.64 (6.33)	3689 (79.64)	16.80 (6.66)
**Did not live with both parents**				
No	652 (66.87)	16.03 (5.69)	3169 (68.42)	16.56 (6.46)
Yes	323 (33.13)	18.41 (7.7)	1463 (31.58)	18.40 (7.79)

Table 2: Mental health opportunity profile

**Rank**	**HH income**	**Mother's education**	**Mother's occupational status**	**Did not live with both parents**	**Group sample size (n)**	**Average k10 score of the participant**	**Risk level**
1	High	Low	High	No	328	16.1	Low
2	High	High	High	No	2032	16.25	Low
3	High	Low	Low	Yes	68	16.53	Low
4	High	High	Low	No	208	16.62	Low
5	High	High	High	Yes	731	17.12	Low
6	Low	High	High	No	231	17.17	Low
7	Low	High	Low	No	100	17.48	Low
8	High	Low	High	Yes	97	17.52	Low
9	High	Low	Low	No	150	17.69	Low
10	High	High	Low	Yes	87	18.72	Low
11	Low	Low	Low	No	81	19.26	Low
12	Low	Low	High	Yes	46	19.28	Low
13	Low	Low	High	No	39	19.97	Low
14	Low	High	High	Yes	185	20.7	High
15	Low	Low	Low	Yes	107	20.89	High
16	Low	High	Low	Yes	142	21.15	High

Table 3: Parameter estimates of different logit regression models
(Depedent variable: Whether likely to have mental disorder- i.e. K10 ≥ 20)

	Null			Logit			Mixed 1			Mixed 2		
**Fixed effects**	Estimate (OR)	Std error	95% CI	Estimate (AOR)	Std error	95% CI	Estimate (AOR)	Std error	95% CI	Estimate (AOR)	Std error	95% CI
Intercept	0.296***	0.059	(0.2-0.437)	0.168***	0.017	(0.138-0.204)	0.075***	0.018	(0.047-0.12)	0.05***	0.011	(0.322-0.078)
Wave (time)				1.019	0.011	(0.998-1.04)	0.999	0.022	(0.957-1.042)	1.00	0.022	(0.961-1.047)
Individual characteristics												
Gender - Female (Ref.: Male)				1.484***	0.108	(1.286-1.712)	2.063***	0.363	(1.461-2.913)	2.021***	0.355	(1.431-2.851)
Rural resident - Yes (Ref. : No)				0.759*	0.095	(0.593-0.97)	0.89	0.195	(0.579-1.366)	0.899	0.197	(0.586-1.383)
Number of financial shock				1.321***	0.041	(1.243-1.404)	1.436***	0.074	(1.298-1.589)	1.412***	0.072	(1.277-1.561)
Number of life event shock				1.156***	0.052	(1.059-1.262)	1.161*	0.081	(1.013-1.331)	1.157*	0.08	(1.01-1.326)
Long term health condition - Yes (Ref. : No)				2.232***	0.212	(1.853-2.688)	2.934***	0.502	(2.098-4.103)	2.855***	0.488	(2.042-3.99)
Club activities - Yes (Ref. : No)				0.651***	0.05	(0.559-0.758)	0.623***	0.078	(0.487-0.797)	0.635***	0.08	(0.496-0.812)
Daily smoker - Yes (Ref. : No)				1.241*	0.125	(1.018-1.512)	1.801**	0.339	(1.246-2.604)	1.676**	0.313	(1.162-2.416)
Heavy drinker - Yes (Ref. : No)				1.344***	0.099	(1.163-1.554)	1.651***	0.209	(1.288-2.117)	1.649***	0.209	(1.286-2.114)
Obese - Yes (Ref. : No)				1.131	0.11	(0.935-1.367)	1.372	0.269	(0.935-2.014)	1.311	0.256	(0.895-1.921)
Background characteristics												
Household Income - Low (Ref. : High)				1.258*	0.116	(1.05-1.506)				1.572*	0.349	(1.017-2.43)
Mothers Education - Low (Ref. : High)				0.972	0.088	(0.814-1.162)				0.921	0.203	(0.597-1.421)
Mothers' occupation - Low (Ref. : High)				1.188	0.109	(0.992-1.423)				1.314	0.296	(0.845-2.043)
Did not live with both parents - Yes (Ref. : High)				1.183*	0.091	(1.017-1.376)				1.586*	0.298	(1.097-2.294)
**Random effects**												
Background (level 3)												
Intercept variance σ^2_{v0}	0.423	0.202	(0.166-1.08)				0.078	0.082	(0.01-0.608)	7.14e-32	3.89e-17	
Individual (level 2)												
Intercept variance σ^2_{u0}	4.101	0.422	(3.353-5.017)				4.068	0.718	(2.878-5.749)	4.116	0.720	(2.921-5.8)
Wave variance σ^2_{u1}							0.062	0.015	(0.039-0.098)	0.062	0.015	(0.039-0.099)
Covariance σ^2_{v0v1}							-0.091	0.071	(-0.231-0.048)	-0.098	0.071	(-0.238—0.041)
ICC												
$rho_{background}$	0.054	0.024	(0.022-0.127)				0.011	0.011	(0.001-0.076)	9.64e-33	5.26e-18	
$rho_{individual \mid background}$	0.579	0.026	(0.527-0.629)				0.558	0.043	(0.472-0.64)	0.556	0.043	(0.47-0.063)

Notes: *** p < 0.001, ** p < 0.01, and * p < 0.05.
