# Supplementary material for: Do family and maternal background matter? A multilevel approach to modelling mental health status of Australian youth using longitudinal data
Source: PLoS One. 2022 Apr 26;17(4):e0267191. doi: 10.1371/journal.pone.0267191 (PMC9041811; doi:10.1371/journal.pone.0267191)
Supplement: Attachment — Submitted filename: response2_rc_pone_v1_221121.docx [file pone.0267191.s003.docx]

**Response to Reviewers’ and Editors’ Comments**

The authors wish to express their sincere appreciation to the anonymous reviewers for their insightful comments and valuable suggestions, which we believe has significantly enhanced the quality of the manuscript. Our responses in reply to each of the comments of the reviewers are listed hereunder.

| Comments | Authors’ Response | Section/ page |
| --- | --- | --- |
| Reviewer 1 |  |  |
| 1. More references to be added to support the introduction, purpose and discussions. Additional comments are attached in the reviewed manuscript. Conclusion should be more specific and can't be generalized. | We thank the reviewer for such valuable suggestions. We have added references and extensively revised the manuscript. Conclusion has been revised for greater clarity. | Whole document |
| 2. Add Maternal | Addressed. Word added. | Keywords, page 2 |
| 3. Suggest to change the word "distributed" (not clear: good mental health distributed?) | Addressed. Deleted the sentence. | Page 3, line 1-3 |
| 4. Suggest to have more harmony between ideas of the first introduction | Addressed. We thank the reviewer. We have harmonised between ideas of the first introduction. | Page 3, line 6-7 |
| 5. Suggest to make this long sentence shorter or divide it into simple sentences. | Addressed. The sentence is shortened. | Page 3, line 12-15. |
| 6. Add age between brackets for this transitioning phase | Addressed. Age band added. | Page 3, line 16-17. |
| 7. Youth circumstances is not clear, suggest to explain a bit what is meant here | Addressed. We thank the reviewer. We have added the type of circumstances within brackets. | Page 3, line 19-20 |
| 8. This should be linked to other studies, the way of saying we do not understand is not clear, the authors do not understand? or others? More references should be checked and mentioned here to confirm the saying. | Addressed. We thank the reviewer. We have refined the sentence and added WHO guideline on improving the early childhood development (2020) and WHO guidelines on mental health promotive and preventive interventions for adolescents (2020) as references. | Page 3, line 22-23 |
| 9. What type of inequalities? this is not well explained. | Addressed. It is mental health inequalities. We have added the term “mental health”. | Page 3, line 25 |
| 10. In general, I believe that additional references are needed to be considered and mentioned, such as WHO Maternal mental health and social determinants of health | Addressed. We have added WHO guideline on improving the early childhood development (2020) and WHO guidelines on mental health promotive and preventive interventions for adolescents (2020) as references. | Page 3, line 2-3 |
| 11. Suggest to change the term: Fill this knowledge gap As one paper may improve the knowledge gap rather than fill it | Addressed. Used the term “improve”. | Page 4, line 1 |
| 12. Suggest to put this at the end of the paper for implications | Addressed. Deleted the sentences. We have put this in the conclusion section. | Page 4, lines 22-24 |
| 13. Any reference for these? | References added. | Page 4, line 17 |
| 14. Reference for figure 1 should be here? | Addressed. | Page 5, line 5 |
| 15. How lifestyle is disregarded however in page 6-line 11-12 health habits are considered such as sporting and hobby? It seems contradictory to me. | We thank the reviewer. For clarification, we've changed the term ‘disregarding’ to ‘controlling’. The theory states that only circumstance variables would need reward through intervention and effort variables would generate reward on its own. Here we mean to control the effort variables (e.g., life style and health habits) so that we can understand the impact of circumstances variables. Addressed the issue. | Page 5, line 18-19 |
| 16. Specify Appendix "A" | The term appendix "A" added. | Page 6, line 9 |
| 17. Explain more here about the unique identifiers | Addressed. The Table 2’s opportunity profile also describes this (rank number). | Page 7, line 7 |
| 18. It seems here as repeated information, suggest to summarize and keep the needed | Deleted repeated sentence. | Page 10, line 17-18 |
| 19. Any reference for this? | Reference added. | Page 10, line 23 |
| 20. This possibility doesn't include a valid reason | We are describing a phenomenon here; and not giving an explanation of such possibilities. Future research is needed to investigate such phenomenon. | Page 11, line 1 |
| 21. To specify these dimensions | Addressed. We have added the dimensions and clarified the sentence. | Page 11, line 18 |
| 22. What about coping strategies for older adults learned from mothers? | Investigating coping strategies are not the purpose of this article. This is an interesting avenue for future research. | Page 11, line 24 |
| 23. To explain more the rational to suggest this particular intervention for policy | Addressed. We thank the reviewer. We have added a line providing examples as to why such intervention policy is needed. | Page 12, line 1 |
| 24. What are these delivery costs? | We thank the reviewer for this query. The existing literature does not indicate the possible delivery cost of these intervention might be. That is why we proposed that such investigations are needed as future research. | Page 12, line 7 |
| 25. To include other findings in line with the introduction and the purpose of the study mentioned before | Addressed. We have added other findings in line with introduction and the purpose of the study. | Page 12, line 9 |
| 26. The conclusion is very broad and should be followed by other specific studies to be able to provide a comprehensive analysis that can be generalized. | Addressed. We thank the reviewer. We have revised the conclusion. | Page 12, line 15 |
| 27. specify ppl what does it stand for | ppl stands for persons. We have changed the words to “persons” for clarity. | Page 17, Figure 1 |
| Reviewer 2 |  |  |
| 1. Mental illness is a leading cause of disability worldwide among young people (aged 10–24 years). Whilst there is a good body of work that has generated substantial evidence about the influence of maternal circumstances on child and adolescent mental health, a key methodological issue in most of them being their exclusive reliance on cross-sectional designs. Longitudinal analyses using repeated measures offer a richer understanding of the natural history as one progress in the life trajectory and can identify risk factors associated with transitions. In this context, the present manuscript on youth mental health using longitudinal data has potential scope for adding further evidence to the existing literature on child and adolescent mental health and will contribute towards life course perspective on mental health problems. However, for better understanding and clarity, I have the following concerns to be addressed: | We thank the reviewer. We believe that our manuscript has been considerably improved following the comments given by the reviewer. We have addressed/clarified your concerns as follows: | N/A |
| 2. English language and grammar used throughout the manuscript needs thorough revision and editing | English language and grammar have been thoroughly revised and the manuscript has been edited by a native English speaker. | Whole document |
| 3. Purpose & Abstract: The method in the Abstract section should go beyond the description of data and provide the method (model, Analysis) used for answering the research question. | We have added a line describing the type of model used in the analysis. | Abstract, line 11-12 |
| Keywords can be better refined to match the appropriate controlled vocabulary | Addressed. Keywords has been refined. | Keywords |
| Wherever possible, a better technical term should be used. For example, “great emphasize or great importance” instead of “great store”; “important determinant/risk factor” instead of socioeconomic status (SES) is an “important marker.” | We thank the reviewer. We addressed this issue. | Abstracts, line 1  /Introduction, 1^st^ paragraph, last sentence. |
| 4. Introduction: As the authors have stated that “circumstances experienced by individuals in their childhood and adolescent period are certainly much different than the period when they are transitioning to youth and adulthood” it is important that how they have addressed or parametrized the time-varying covariates in their model. Currently, the manuscript falls short of providing the detail. | We thank the reviewer for this query. We have specifically adopted the random effect multilevel logistic regression model to handle time varying components. The time varying component has been handled in the random effects component. This has been discussed in the statistical analyses and regression analysis section. Readers can find Table 3 random effects panel for wave variance to check the effects of time varying components. | Statistical analyses, line 9-10, page 8; regression analysis, page 10 |
| Also, it would be added value to show how the covariates have varied over time to substantiate the above statement. | We thank the reviewer for this query. This has been discussed in the statistical analyses and results section. The estimates of the time varying component can be seen in the random effects panel of Mixed 1 and Mixed 2 model of Table 3. | regression analysis, page 10, Table 3 |
| 5. In the term “to investigate the impact of youth circumstances”, circumstances here mislead one to assume those recent or current occurrences around youth period. They may not be appropriate to describe the maternal circumstances. The authors need to use better term to denote the maternal circumstances.  The specific objective or research question, or hypothesis must be mentioned explicitly besides the primary goal. Otherwise, it will allow readers to make their own assumptions. | Addressed. We have included examples about what youth circumstances are considered here. We have not considered maternal circumstances in our study. Here we used maternal background characteristics as one of the youth circumstances. As per suggestion, we have clarified the term as “maternal background groups”.  We have added specific research questions for clarity. | Introduction, page 4 |
| 6. Methods: The authors should have clarity and consistency in using the terms mental health problems, mental health distress and mental disorders. Mental disorders usually require a diagnostic tool. K10 is used to detect non-specific psychological distress, not mental disorders. | We thank the reviewer for this query. It has been widely recognised in the literature in a population wide setting that K10 (Kessler 10-point scale) can be used as a screening tool for mental disorder. This is because K10 scale has a high correlation with the likelihood of having mental disorder. Supporting references can be found in the following research:  1. “Kessler, RC, Barker, PR, Colpe, LJ, Epstein, JF, Gfroerer, JC, Hiripi, E, Howes, MJ, Normand, S-LT, Manderscheid, RW, Walters, EE & Zaslavsky, AM 2003, 'Screening for Serious Mental Illness in the General Population', Archives of General Psychiatry, vol. 60, no. 2, pp. 184-9.”  2. “Andrews, G & Slade, T 2001, 'Interpreting scores on the Kessler Psychological Distress Scale (K10)', Australian and New Zealand Journal of Public Health, vol. 25, no. 6, pp. 494-7”. | Outcome variable, exposure variables and other co-variates section, page 6. |
| 7. The authors do not specify the point in time at which the covariates for maternal background status are considered. Are these the data collected at baseline? If so, how would they take into account of time-varying components? Household income might change over time. For other covariates, are baseline values used? Time-varying values? Values from the previous period? | We thank the reviewer for this query. Maternal background status has been determined at the baseline wave 7. As discussed before all-time varying component has been addressed through our modelling technique and this is one of our chief contributions. We used random effect multilevel mixed effect logistic regression to addressed time varying components. The estimates of the time varying component can be seen in the random effects panel of Mixed 1 and Mixed 2 models of Table 3. | Statistical analyses, page 8; regression analysis, page 10, Table 3. |
| 8. For demographic covariates, there are many families that move from rural to urban, and some vice versa. How this was addressed in the analyses. | We thank the reviewer for this query. We have added rural resident dummies to address this phenomenon. The random effects are estimated in the random effects panel where this variance is accounted for. | regression analysis, page 10, Table 3. |
| 9. Authors need to provide brief details on how they have addressed repeated measures, multi-collinearity and interaction -effects in their model. Probably, the author can bring more clarity to the nested structure through diagrammatic illustration. Further, instead of “maternal background history” the author should use “maternal background history groups”, as it allows one to assume it as an individual characteristic without the qualifier. | Addressed. We thank the reviewer for this suggestion. We have added the diagrammatic illustration. We also modified the term maternal background history” to “maternal background history groups”. | Page 8, line 10-11 |
| 10. Results: The results section appears a little terse, and the language used to describe the results needs to be reconciled for better clarity. For example, one loses clarity on the sentence “All groups have approximately six to seven points of standard deviation which indicates considerable variability at the individual level”; it was unclear what the authors meant by “all groups” and how group value indicates individual variability. This information can be added in Table 1 for better clarity. | We thank the reviewer for the suggestion. We have rewritten the sentence for better clarity. | Page 9, line 13-20. |
| 11. The authors can prefer to use the term “substantially” instead of significantly in the sentence “It can be seen that age groups do not vary significantly in mean K10 score both”, as it was not finding from statistical analyses. | Agreed. We thank the reviewer for this suggestion. We have modified the sentence accordingly. | Page 9, line 5 |
| 12. Though not a statistical expert, it was not clear either from their description of their statistical analyses or from table 3 how they arrived at individual estimates for background characteristics if they were grouped to form level 3 with 16 groups. Suppose the background characteristic were entered at the individual level (2), then how they can be a component of level 3. For example, if maternal education is one component that contributed to form level 3 group, then how one can derive estimates for maternal education. The authors need to specify which variables corresponded to random slope and random intercept. | We thank the reviewer for this query. As can be seen in Table 2, and explained in “Outcome variable, exposure variables and other co-variates” and statistical analyses section, the groups are defined by a combination of the background characteristic variables. This combination is explained by the rank number of Table 2. Thus, the rank number of Table 2 explains the level three group variations of Table 3. Each of the groups level variables (HH income, Mothers education and occupation, living arrangement) then can enter as fixed effects in Mixed 2 model, which is shown in Table 3. Thus, in summary with the reviewer example, maternal education is the component that contributed the rank number of the group variable. Thus, in the final analysis the rank number is explaining level 3, which is a separate variable itself. So, naturally individual group level characteristic variables now can enter in level two as separate variables. | Statistical analyses, page 8 |
| 13. The components like household income, family living arrangement, maternal education and occupational status are very much a characteristic at individual level. However, the authors have used these components and formed 16 subjective groups. In that sense, I presume that there was no natural nesting. The authors need to provide strong rational for artificially creating a group and its practical relevance. | We thank the reviewer for this query. One of the hypotheses of our paper is to check whether there are maternal background level variations. To check this hypothesis, we have constructed this groups. In summary this groups were created to test the hypothesis whether maternal background matters and if so, then at what level (or if not, then at what level)? Thus, we created this group as per our research question and to answer this question, we did not find any group level variation. This is a very important contribution of our research. | N/A |
| 14. Discussion: Overall, the discussion needs strengthening by relating the results with the available literature and include strengths and limitations. The authors need to discuss the limitation of dichotomizing the categorical outcome especially in the context that increase in K10 score signifies increase in distress. | We thank the reviewer. We have added a limitation paragraph in our discussion section. | Discussion, page 15. |
| 15. The way the group (level 3) was conceptualized has a significant impact on the study finding; there are evidences linking maternal characteristics with some individual characteristics which were significantly associated with mental health of young. Without looking at moderation/interaction effects, any conclusion on maternal characteristic would be unconvincing. | As discussed before, level 3 groups were conceptualised to test the hypothesis whether background characteristics matters at level 3. That is why we proposed four models in Table 3: Null model, traditional logit model Mixed 1 model without background characteristics at level 2 with level 3 variation and Mixed 2 model with background characteristics at level 2 with level 3 variation. Once fixed effect background characteristics is considered the level 3 effects vanishes in mixed 2 model. Even in traditional logit model and mixed 1 model maternal background characteristics effects were not strong. Using all these model sensitivity parameters we arrived at very strong conclusions. | N/A |
