# Supplementary material for: Do family and maternal background matter? A multilevel approach to modelling mental health status of Australian youth using longitudinal data
Source: PLoS One. 2022 Apr 26;17(4):e0267191. doi: 10.1371/journal.pone.0267191 (PMC9041811; doi:10.1371/journal.pone.0267191)
Supplement: Attachment — Submitted filename: response_rc_plos_300322.docx [file pone.0267191.s004.docx]

**Response to Reviewers’ and Editors’ Comments**

The authors wish to express their sincere appreciation to the anonymous reviewers for their insightful comments and valuable suggestions, which we believe has significantly enhanced the quality of the manuscript. Our responses in reply to each of the comments of the reviewers are listed hereunder.

| Comments | Authors’ Response | Section/ page |
| --- | --- | --- |
| **Reviewer #2** |  |  |
| Overall, the authors have addressed most of the concerns. However, based on the response by the  authors, I have the following concerns: | We thank the reviewer for such valuable suggestions. |  |
| 1. The authors should remove the term “group” from “maternal background group” in all the places except in the statistical analyses section. It would not be appropriate and easily comprehensible to use the term “maternal background group” in all the places. | Addressed. We thank the reviewer. | Whole document except for the statistical analysis section. |
| 2. As the grouping involves HH income and family living arrangement, which are more of a family background characteristic, it would be misleading and erroneous to include and label it as maternal background characteristics. The face validity of the construct of maternal background becomes debatable when HH income and family living arrangement, which are more of a family background characteristic, were included to define the maternal background characteristics. The authors need to use a more valid and appropriate term as it has implications for the conclusions. This is particularly important when this paper makes a strong claims and conclusions about the lack of impact of maternal background on youth mental health.  In this context, the authors need to refine the title to reflect the content and context more accurately. | Agreed. We appreciate the reviewer bringing this to our attention. Rather than referring to the group's characteristic as just maternal, we now refer to it as the 'family and maternal background.' As suggested, we also modified our title as “Do family and maternal background matter? A multilevel approach to modelling mental health status of Australian youth using longitudinal data”. Addressed. | Whole document |
| 3. From the author's response, it became evident that the grouping was forced artificially to answer the research  question, and the model did not follow natural nesting. The very nature of grouping and the subsequent modelling and conclusions does not rule out the possibility of ecological fallacy completely. This will be a serious concern, and the authors need to be cautious in making strong conclusions on the lack of impact of maternal background on youth mental health. Accordingly, the abstract and discussion section should be refined and revised further to account for the limitations of the grouping and modelling. | We formulated the groups with the null hypothesis that there is no group level variation and checked whether we can reject this null. Since it is almost impossible that a nationally representative dataset would nest the data set the way we want, we designed our study this way to answer our research question. We also accommodated individual level variations to account for any additional variation through individual level characteristics in our research design. However, for our sample, there was not enough evidence to reject the null.  We agree that this does not rule out the possibility of ecological fallacy completely. For this reason, we acknowledged this limitation in the revised discussion section. Addressed. | Page 16 paragraph 2 |
